# Hydraulic limitation drives differential crown dieback in *Populus alba* varieties

**DOI:** 10.48130/forres-0026-0017

**Published:** 2026-05-15

**Authors:** Shu-Dian Liu, Dong-Dong Luo, Geng-Yun Zhao, Yue-Xia Yu, Jun-Tuan Zhai, He Wang, Zhi-Chao Xia, Li-Dong Fang

**Affiliations:** 1 Anhui Provincial Key Laboratory of Forest Resources and Silviculture, School of Forestry and Landscape Architecture, Anhui Agricultural University, Hefei 230036, China; 2 State Key Laboratory Incubation Base for Conservation and Utilization of Bio-Resource in Tarim Basin, Key Laboratory of Protection and Utilization of Biological Resources in Tarim Basin of Xinjiang Production & Construction Corps, College of Life Science and Technology, Tarim University, Alar 843300, China; 3 Wanzhong Comprehensive Experimental Station, Anhui Agricultural University, Hefei 231521, China

**Keywords:** *Populus* decline, Hydraulic architecture, Non-structural carbohydrates, Tree-ring width index, Drought adaptation

## Abstract

The widespread decline and mortality of the Three-North Shelter Forest under climate change is threatening the sustainability of arid and semi-arid ecosystems. As the dominant tree genus in these shelterbelt forests, poplars (*Populus* spp.) display clear varietal differences in decline patterns, yet the underlying physiological mechanisms remain poorly understood. To address this question, we compared two poplar varieties, *Populus alba* var. *bachofenii* (*P.b*) and *Populus alba* var. *pyramidalis* (*P.p*), using branch samples from upper and lower canopy positions. The results showed that although hydraulic conductivities (*K*_*s*_ and *K*_*l*_) did not differ significantly between varieties, *P.b* consistently exhibited lower percent loss of conductivity (PLC), particularly in the upper canopy branches. In contrast, *P.p* experienced more negative midday water potentials (*Ψ*_md_) in the upper crown, indicating greater water deficit. Within *P.p*, declining individuals (*P.p-D*) displayed higher PLC, whereas hydraulic conductivity, predawn water potential (*Ψ*_pd_), and non-structural carbohydrates (NSC) concentrations were similar to those of healthy trees, indicating that dieback was associated primarily with increased hydraulic vulnerability, rather than reduced transport capacity or carbon depletion. Tree-ring analyses further revealed stable growth in *P.b*, whereas *P.p* was more drought-sensitive and showed greater interannual variability. Trees with higher PLC exhibited greater growth sensitivity and reduced radial growth rates, supporting the hydraulic limitation hypothesis. Overall, this study clarifies the physiological mechanisms underlying the contrasting decline patterns among poplar varieties in water-limited regions, and provides guidance for optimizing species selection and management strategies in arid shelterbelts.

## Introduction

In recent decades, the frequency and intensity of extreme drought events has increased, causing widespread forest degradation and tree mortality worldwide; a trend that is expected to intensify with future climate warming^[1]^. Climate-driven disturbances can substantially reshape forest ecosystems by altering stand structure, modifying species composition and distribution, and weakening key ecosystem functions^[2]^. In arid and semi-arid regions, water availability is a key limiting factor for tree growth^[3]^, making these water-limited systems increasingly vulnerable to drought-induced mortality^[4]^. This knowledge is therefore critical for improving predictions of climate-driven shifts in water-carbon dynamics and for developing more effective forest management and climate adaptation strategies^[5]^.

Tree mortality under drought stress is generally attributed to two main mechanisms: hydraulic failure and carbon starvation. According to the hydraulic failure hypothesis, when xylem water potential falls below a critical threshold, air is drawn into conduits through intervessel pits, initiating cavitation and embolism that disrupt the continuity of water transport^[6]^. This breakdown of hydraulic function is now regarded as one of the primary drivers of drought-induced decline and mortality in trees^[7,8]^. By contrast, the carbon starvation hypothesis emphasizes that early stomatal closure and sustained reductions in stomatal conductance limit carbon assimilation via photosynthesis, causing a gradual depletion of non-structural carbohydrate (NSC) reserves and potentially triggering carbon starvation^[9,10]^. Collectively, these physiological and ecological factors interact to determine tree responses to drought stress^[11]^.

Among temperate woody species, poplars are among the most susceptible to drought-induced xylem embolism^[12]^. A global meta-analysis of 475 plant species showed that hydraulic traits are the most effective predictors of plant mortality, explaining approximately 70% of the observed mortality rates^[13]^. In *Populus*, accumulating evidence indicates that drought-induced decline is typically initiated by hydraulic dysfunction, which often precedes substantial depletion of NSC reserves^[4,14]^. Although drought stress can modify the concentration and allocation of NSC within *Populus* tissues, carbon-related adjustments typically play a secondary role relative to hydraulic failure^[15]^. Previous studies have revealed considerable variation among *Populus* varieties in xylem structure and embolism resistance. In addition, both hydraulic traits and NSC allocation may vary along vertical canopy gradients within individual trees^[12,16]^. However, how water transport and carbon allocation are coordinated along canopy gradients in co-occurring *Populus* varieties remains incompletely understood, limiting our ability to explain their observed contrasting decline patterns.

As trees grow taller, water transport from the roots to the upper canopy must overcome greater gravitational forces and increasing hydraulic resistance, thereby reducing xylem conductivity and steepening the water potential gradient^[17,18]^. Consequently, tissues located in the upper canopy are often exposed to greater hydraulic tension and are therefore more susceptible to water deficit and cavitation^[19]^. Across, and within species, taller trees tend to be more vulnerable to drought, often exhibiting a characteristic 'top-down' dieback pattern in their shoots^[10]^. This phenomenon aligns with the 'hydraulic limitation hypothesis', which links taller tree height to reduced hydraulic efficiency and top-down canopy dieback^[20]^. To mitigate water deficits, trees often shed their upper leaves and branches. However, species and organs differ in their capacity to make these adjustments, depending on the coordination between hydraulic function and carbon reserves.

Tree-ring width (TRW) provides a long-term and high-resolution archive of radial growth, reflecting trees' responses to environmental variability over time^[21]^. In shelterbelt plantations established in arid and semi-arid regions, tree growth depends primarily on irrigation and groundwater recharge rather than on natural precipitation alone^[22]^. Even under managed irrigation regimes, spatial heterogeneity in groundwater depth, irrigation frequency, and root access to deeper soil moisture can generate substantial variability in tree water status and vitality^[23]^. Unlike natural forests, the decline of plantation trees is therefore shaped not only by regional climatic conditions, but also by local water management practices and irrigation regimes^[24]^. Long-term irrigation can also alter below-ground conditions, potentially imposing additional constraints on tree performance^[25]^. Previous studies have shown that shallow groundwater regulates transpiration in shelterbelt forests^[22,26]^, whereas deep soil water deficits accelerate the degradation of *Populus* plantations. Notably, even under irrigation, some individuals exhibit apical dieback and reduced vigor, suggesting that internal hydraulic dysfunction or constraints on carbon allocation may underlie the observed decline patterns^[10,27]^. Within this context, TRW serves not only as an indicator of tree water status and growth reduction, but also as a valuable early signal of forest degradation^[28]^. Therefore, integrating TRW with hydraulic traits enables the assessment of both short-term drought impacts and long-term growth trajectories^[29]^.

Xinjiang, located in arid northwest China, is characterized by severe droughts threatening local forests and plantations^[2,30]^. Fast-growing poplar*s* are widely planted in the Three-North Shelterbelt because of their rapid establishment and strong adaptability^[31]^. Among them, *Populus alba* var. *bachofenii* (*P.b*) and *Populus alba* var. *pyramidalis* (*P.p*) play key roles in windbreak establishment, sand stabilization, and ecological restoration. However, unlike the relatively healthy *P.b*, many *P.p* individuals exhibit apical dieback, reduced vigor, and premature leaf shedding, particularly in the upper crown^[4]^. Despite increasing recognition of hydraulic limitation and carbon starvation as mechanisms of drought-induced decline, it remains unclear whether intervarietal differences in crown dieback within *Populus alba* are primarily driven by hydraulic vulnerability or by carbohydrate depletion. In addition, a few studies have integrated canopy-position-specific hydraulic traits with long-term tree-ring growth to mechanistically explain these decline patterns. We investigated healthy individuals of *P.b* and both healthy and declining individuals of *P.p (P.p-D)* in shelterbelt plantations in Alar City, Xinjiang. Measurements included predawn (*Ψ*_pd_) and midday (*Ψ*_md_) leaf water potentials, branch hydraulic traits, NSC concentrations across canopy positions, and tree-ring width indices (RWI). We aimed to: (i) investigate how *P.b* and *P.p* coordinate water transport and carbon storage along canopy height gradients under drought stress; (ii) test whether hydraulic limitation contributes to canopy decline in *P.p*; and (iii) examine whether combining short-term hydraulic traits with long-term tree-ring growth records can help identify intervarietal differences in mortality risk. Based on the contrasting growth performance and canopy dieback observed at our study site, and a potential growth-hydraulic safety trade-off between the two varieties, we formulated two hypotheses: (i) intervarietal differences in drought vulnerability between *P.b* and *P.p* arise from divergent plastic responses of branch functional traits along the vertical canopy gradient, whereby insufficient hydraulic coordination in the upper canopy of *P.p* predisposes branches to dieback; (ii) a trade-off exists between growth and hydraulic safety in the two poplar varieties, with *P.p* favoring higher growth efficiency at the cost of increased embolism risk; this trade-off should be reflected in both short-term hydraulic traits and long-term tree-ring growth patterns.

## Materials and methods

### Study site and plant materials

**Figure 1:**
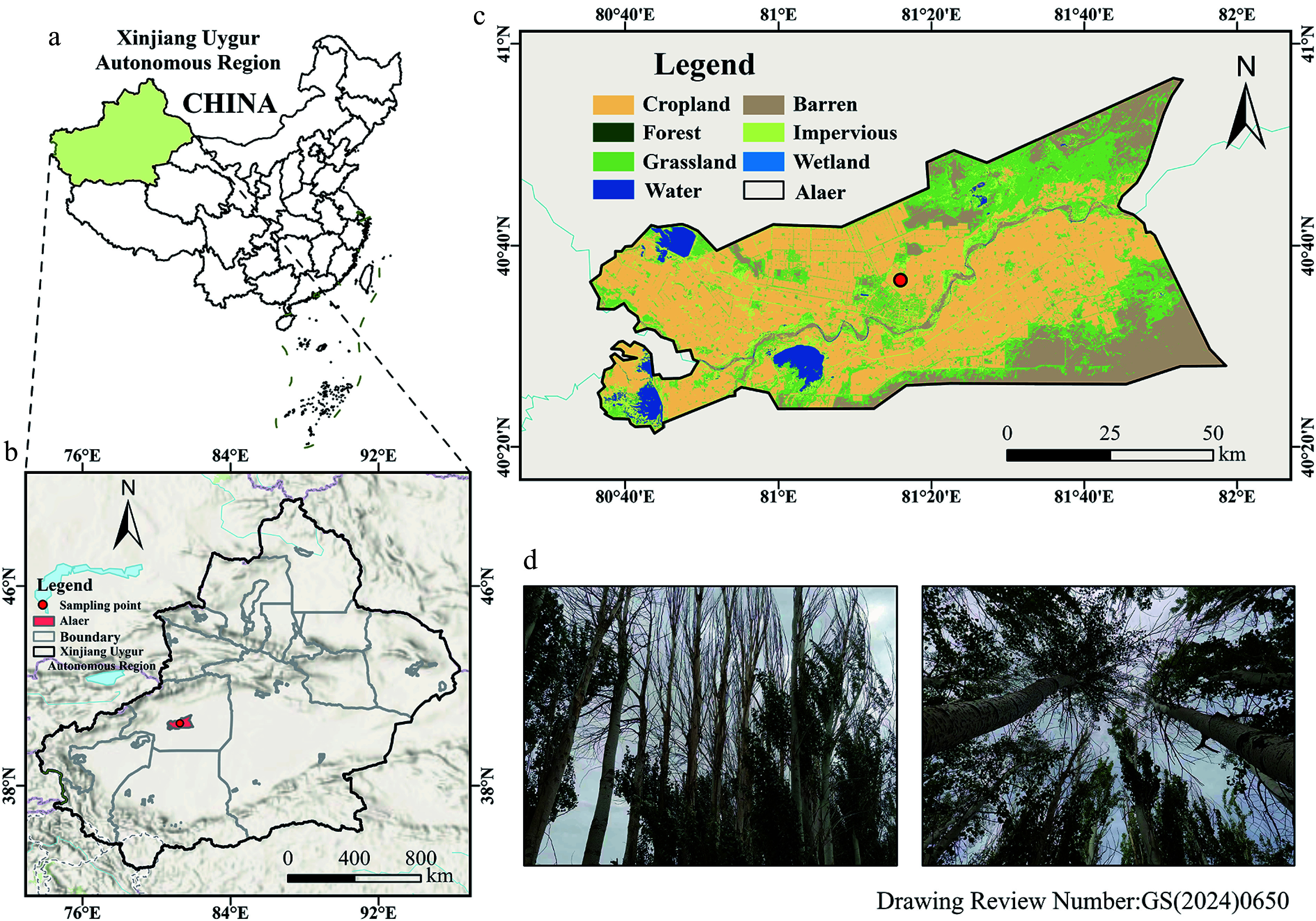
Map of the study area. (a) Location of the Xinjiang Uygur Autonomous Region in China. (b) Detailed location of Alar City in Aksu Prefecture, with the red dot indicating the specific study site. (c) Land use classification in Alar City, where dark green denotes forested regions and brown indicates agricultural land, both featuring farmland protection forests established to combat wind erosion and desertification. (d) Photograph showing farmland shelterbelts with poplars exhibiting varying degrees of decline. Base maps (a–c): https://cloudcenter.tianditu.gov.cn/administrativeDivision.

Alaer City is a county-level city in the Xinjiang Uyghur Autonomous Region of northwestern China (80°30′–81°58′ E, 40°22′–40°57′ N), located on the southern slope of the Tianshan Mountains and along the northern border of the Taklimakan Desert. Our specific sampling site (marked by the red dot in Fig. 1b, c) is positioned at 81°18′ E, 40°36′ N. The region has an annual precipitation of 40.1 to 82.5 mm, and an annual evaporation ranging from 1,876.6 to 2,558.9 mm^[32]^. Under these extremely arid conditions, precipitation contributes little to soil water supply, and groundwater levels are mainly sustained by agricultural irrigation, a typical feature of farmland shelterbelt systems in this region^[22]^. The study area is part of the Tarim River alluvial plain, featuring severe desertification, salinization, and soil degradation, resulting in limited natural forest coverage^[33]^.

Field surveys conducted in local farmland shelterbelts indicated that *P.p*, the dominant planted species in the region, frequently displayed dieback symptoms in the upper canopy. In contrast, comparable signs of decline were not observed in *P.b* (Fig. 1d). To investigate the physiological mechanisms underlying this contrasting canopy performance, branch samples were collected during July 2021 (peak growing season). Samples were carried out across different canopy positions in three tree groups: healthy *P.b*, healthy *P.p*, and declining *P.p-D* individuals, with six trees per group (*n* = 6). All sampled trees originated from plantations established in the same year, ensuring comparable stand age among the groups. Mean diameter at breast height (DBH), estimated from cumulative TRW measurements, was 22.54 cm for *P.b*, 26.46 cm for *P.p*, and 22.62 cm for *P.p-D*, with tree height ranging from 18 to 22 m. Maximum vessel length was determined using the air-injection technique at 60 kPa following the method described by Melcher et al.^[34]^, with mean values of 31.16 cm in *P.b* and 36.33 cm in *P.p*. Trees were planted at a spacing of 2 × 2 m as part of windbreak and sand-fixation shelterbelts. All sampled individuals grew under similar environmental conditions (comparable soil properties, groundwater depth, irrigation regimes, and planting density), minimizing environmental influences on hydraulic traits and growth. To characterize physiological differences between the two varieties, we measured hydraulic traits and quantified NSC concentrations in multiple tissues. In addition, tree RWI data were combined with radial growth rates to evaluate links between short-term hydraulic traits and long-term growth dynamics.

### Leaf water potential

For each group (*P.b, P.p*, and declining *P.p-D*), six representative sun-exposed twigs were sampled from both upper and lower canopy positions. Immediately after sampling, the twigs were sealed in Ziploc bags containing moist paper towels to maintain hydration and then transported to the laboratory without delay. Leaf water potential was determined using a pressure chamber (PMS 1505D; PMS Instrument Company, Albany, OR, USA; in MPa). Measurements were performed during two periods representing the daily extremes of plant water status: predawn (04:00–06:00) and midday (12:00–14:00).

### Hydraulic traits

Before sunrise, six sun-exposed branches (~50 cm in length) were sampled from both upper and lower canopy positions of *P.b, P.p*, and declining *P.p-D*. Immediately after sampling, the branches were submerged in buckets containing distilled water, covered with black plastic bags to reduce transpiration, and transported to the laboratory. Hydraulic conductivity was measured on stem segments excised underwater to avoid additional embolism. Stem segments approximately 20 cm in length were used for the measurements. Initial hydraulic conductivity (*K*_*h*_, kg·m^−1^·s^−1^·MPa^−1^) was determined using a 50 cm water head pressure with degassed 20 mmol·L^−1^ KCl solution, and was calculated according to the following equation:

1 $ K_{\mathrm{\mathit{h}}}=\dfrac{J_v}{\Delta\mathrm{P}/\Delta\mathrm{L}} $

where, *J*_*v*_ (kg·s^−1^) is the flow rate and ΔP/ΔL (MPa·m^−1^) is the pressure gradient. Following the initial conductivity measurement, the stem segments were flushed with degassed 20 mmol·L^−1^ KCl solution at 0.1 MPa for 15−20 min to remove xylem embolisms. After this flushing procedure, maximum hydraulic conductivity (*K*_h-max_, kg·m^−1^·s^−1^·MPa^−1^) was determined. The percent loss of conductivity (PLC) was subsequently calculated using the following equation:

2 $ \mathrm{PLC}=\dfrac{100\left({K}_{h-\max }-{K}_{h}\right)}{{K}_{h-\max }} $

For anatomical analyses, basal stem segments (10 cm in length) were perfused with a 0.1% basic fuchsin solution^[35]^. Transverse sections were subsequently scanned using an EPSON scanner and analyzed with ImageJ software to quantify sapwood area (SA, mm^2^) and the total cross-sectional wood area (WA, mm^2^). All leaves attached to the measured stems were scanned to determine total leaf area (LA, m^2^). The leaves were then oven-dried at 105 °C for 30 min, followed by drying at 75 °C for 48 h until a constant dry mass was reached (LM, g). From these measurements, two structural ratios were derived: SA/WA (mm^2^·mm^-2^), and LA/SA (m^2^·mm^−2^). Hydraulic conductivity was normalized by SA and LA following Mencuccini et al.^[36]^. Specific conductivity (*K*_s_, kg·m^−1^·s^−1^·MPa^−1^) and leaf-specific conductivity (*K*_l_, kg·m^−1^·s^−1^·MPa^−1^) were calculated using the following equations:

3 $ {K}_{s}=\dfrac{{K}_{h}}{\mathrm{SA}} $

4 $ {K}_{l}=\dfrac{{K}_{h}}{\mathrm{LA}} $

### Xylem anatomical traits

Wood density (WD, g·cm^−3^) was determined using six replicate stem segments (2−3 cm in length) collected from the basal portion of the branches. After removing the bark, the xylem samples were submerged in water for 24 h to ensure full saturation. Fresh volume was then measured using the water displacement method after gently blotting surface moisture. Subsequently, the samples were oven-dried at 65 °C until constant mass was achieved. WD was calculated as the ratio of dry mass to fresh volume following Hacke et al.^[37]^. After hydraulic conductivity measurements, transverse sections (20 μm thick) were prepared from the distal end of each stem using a semi-automatic microtome (Leica, Germany). The sections were temporarily stained with 0.1% methylene blue to enhance the visibility of xylem anatomical structures. For each stem, two sections were imaged at magnifications of 10× and 40× vessel diameter (D, μm) and vessel density (VD, mm^−2^) were subsequently quantified using ImageJ software.

### Non-structural carbohydrates (NSC)

Samples of leaves, xylem, and bark used for NSC analysis were collected from the same branches that had been used for hydraulic trait and biomass measurements. After collection, the samples were oven-dried at 105 °C for 30 min, followed by further drying at 75 °C for 48 h until constant mass was obtained. The dried tissues were then ground to a fine powder using a JXFSTPRP-64L ball mill and stored in sealed bags prior to analysis. Soluble sugars and starch were quantified using the anthrone colorimetric method^[38]^. Soluble sugars were extracted with 80% ethanol, whereas starch was extracted from the remaining residue using distilled water and perchloric acid. Absorbance was measured at 620 nm with a UV SP-752 spectrophotometer (Shanghai Techcomp Bio-equipment Ltd., China). Total NSC concentration was calculated as the sum of soluble sugars and starch and expressed as mg·g^−1^ dry mass.

### Chronology development and estimation of cumulative basal area increment

Increment cores were collected at breast height (1.3 m) from 10 trees per group *P.b*, *P.p*, and declining *P.p-D* trees, with two cores per tree. After air-drying, the cores were mounted on wooden holders with adhesive and progressively polished with increasingly fine sandpaper to clearly expose annual growth rings for measurement^[21]^. Ring widths were measured to a precision of 0.001 mm using a LINTAB measuring system. Cross-dating was verified with the COFECHA program to ensure chronological accuracy^[39]^. Cores showing decay, structural defects, or correlations below the 95% threshold with the reference chronology were excluded from further analysis. To isolate climate-related growth signals, ring-width series were detrended using the ARSTAN program with age-related curve fitting^[40]^, thereby removing ontogenetic growth trends and other non-climatic influences.

Radial growth rates among the three groups were compared using cumulative basal area (CBA) increment as the growth indicator. In this analysis, tree age was treated as the independent variable in a linear regression model. CBA was derived from raw, non-detrended TRW data according to the following equation^[41]^:

5 $ \mathrm{CB}{A}_{i}=\pi r_{i}^{2} $

where, CBA_*i*_ is the cumulative basal area in *i*-^th^ year, and *r_i_* is the cumulative radius from the 1^st^ to the *i*-^th^ year. For each group, CBA values were first calculated for individual trees and then averaged to construct a mean CBA chronology. The resulting mean series was subsequently regressed against tree age using a linear regression model. The slope of this regression was used to represent the average radial growth rate. In this context, a steeper slope indicates a higher growth rate, whereas a lower slope corresponds to slower radial growth.

### Statistical analyses

Given the observed differences in decline patterns between upper and lower canopy branches of *P.b* and *P.p*, branch samples were collected separately from the upper and lower canopy layers for analysis. For intervarietal comparisons, only sun-exposed branches from the mid-to-upper canopy were selected to minimize light heterogeneity. Differences were assessed at three levels: intervarietal (*P.b* and *P.p*), intravarietal (healthy *P.p* and declining *P.p-D*), and among canopy positions.

For both intervarietal and intravarietal datasets, two-way ANOVA was used to test the effects of group (variety or health status), canopy position, and their interaction. When interactions were significant (*p* < 0.05), simple effects analyses were conducted to examine group differences within each canopy position and vertical differences within each group. When interactions were non-significant, main effects were interpreted, and post hoc comparisons were performed using Tukey's HSD test. Data satisfied the assumptions of normality and homogeneity of variance. For significant main effects, stratified comparisons were conducted to identify biologically meaningful patterns: group effects were evaluated within the upper canopy, and canopy position effects were evaluated within each group. All statistical analyses and graphical visualizations were performed using R software (v4.5.0).

## Results

### Leaf water status

**Figure 2:**
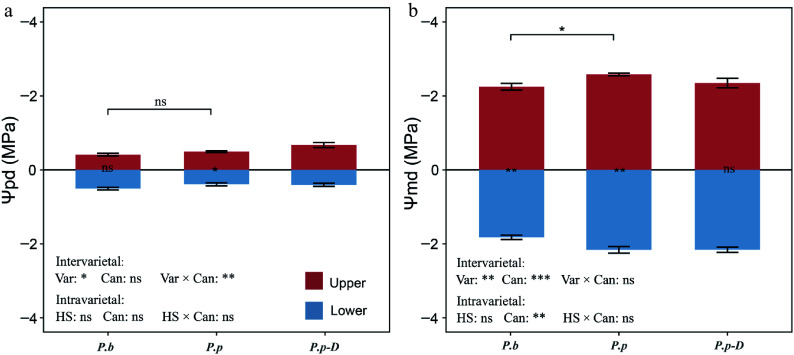
Variations in (a) *Ψ*_pd_ (predawn leaf water potential), and (b) *Ψ*_md_ (midday leaf water potential) across different comparisons: intervarietal (*P.b* vs *P.p*), intravarietal (*P.p* vs declining *P.p-D*), and between upper and lower canopy layers within each group. The effects of variety (or health status) and canopy position were assessed using two-way ANOVA. In the panel annotations, bracketed labels show between-group differences; bar-side labels show within-group canopy differences. 'Var' denotes variety, 'HS' denotes health status, 'Can' denotes canopy position, and 'Var × Can/HS × Can' denotes their respective interactions. 'ns' indicates no significant difference; asterisks indicate significant differences (* *p* < 0.05, ** *p* < 0.01, *** *p* < 0.001). Data are means ± SE (*n* = 6).

Leaf water potential showed clear variation along the canopy gradient, and the magnitude of this variation differed between *Populus* varieties (Fig. 2). In the comparison between *P.b* and healthy *P.p*, *Ψ*_pd_ was significantly influenced by variety (*p* < 0.05) as well as by the interaction between variety and canopy position (*p* < 0.01; Fig. 2a). No significant difference in *Ψ*_pd_ was detected between the two varieties in the upper canopy (*p* > 0.05). Within healthy *P.p* individuals, however, *Ψ*_pd_ was significantly lower in the upper canopy than in the lower canopy (−0.49 vs −0.30 MPa; *p* < 0.05). Such a vertical difference was not observed in *P.b* (*p* > 0.05). Midday water potential (*Ψ*_md_) was significantly affected by both variety (*p* < 0.05) and canopy position (*p* < 0.001), while the interaction between these factors was not significant (Fig. 2b). Overall, *Ψ*_md_ values were more negative in *P.p* than in *P.b* (−2.58 vs −2.25 MPa; *p* < 0.01). In addition, both varieties exhibited a clear canopy gradient, with lower *Ψ*_md_ values in the upper canopy compared with the lower canopy.

Within *P.p*, comparisons between healthy and declining *P.p-D* revealed no significant effects of health status, canopy position, or their interaction on *Ψ*_pd_ (*p* > 0.05; Fig. 2a), indicating broadly similar predawn water status across health conditions and canopy levels. In contrast, canopy position significantly affected *Ψ*_md_ (*p* < 0.01), whereas health status and the interaction term were not significant (Fig. 2b). Healthy *P.p* trees showed a significant vertical gradient in *Ψ*_md_, with more negative values in the upper canopy than in the lower canopy (−2.58 vs −2.16 MPa; *p* < 0.05). This canopy-related difference was not evident in declining *P.p-D*.

### Hydraulic architecture and xylem anatomical traits

**Figure 3:**
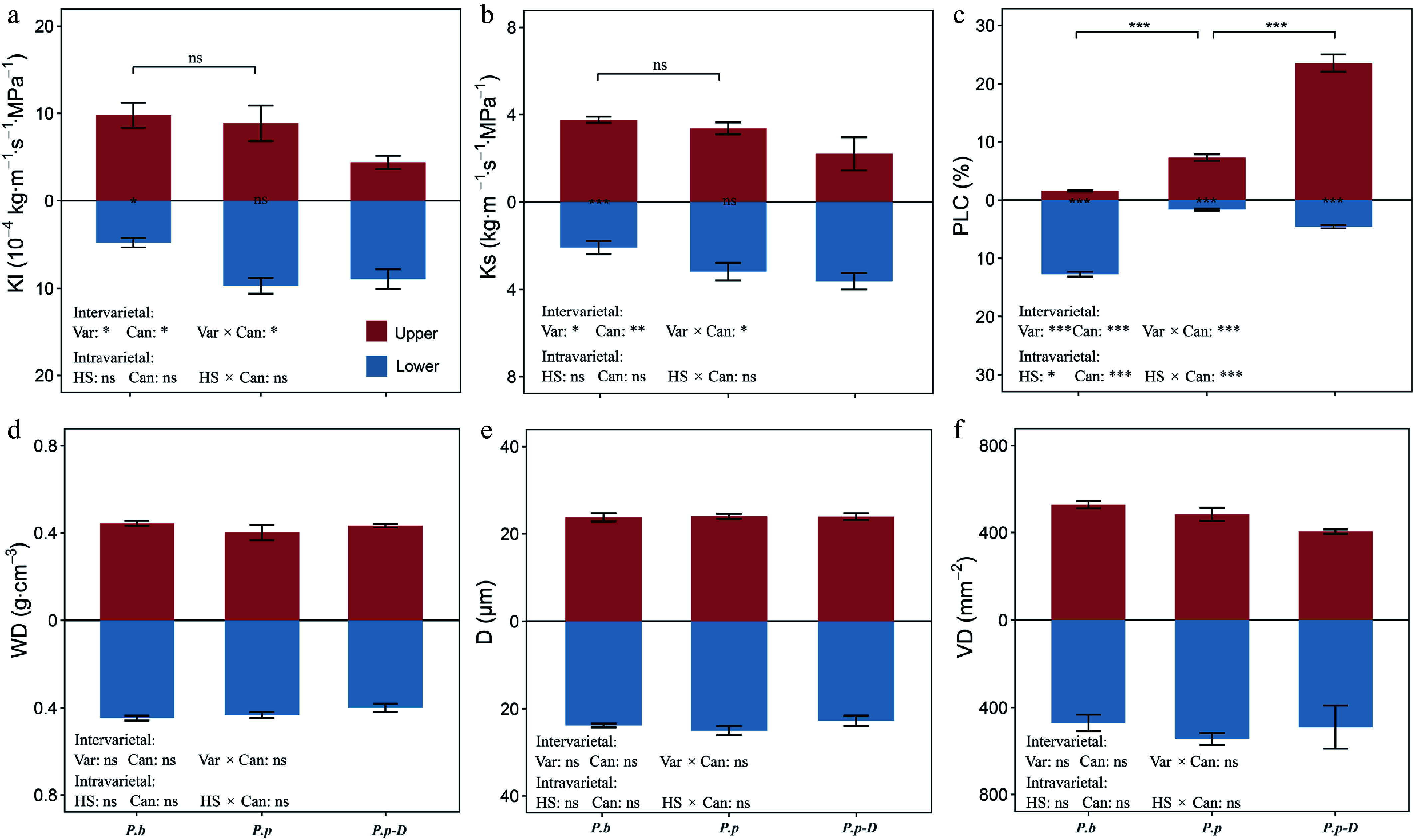
Variations in (a) *K*_*1*_ (leaf-specific hydraulic conductivity), (b) *K*_*s*_ (sapwood-specific hydraulic conductivity), (c) PLC (percent loss of hydraulic conductivity), (d) WD (wood density), (e) *D* (mean vessel diameter), and (f) VD (vessel density) across different comparison groups: intervarietal (*P.b* vs *P.p*), intravarietal (healthy *P.p* vs declining *P.p-D*), and between upper and lower canopy positions within each group. The effects of variety (or health status) and canopy position were assessed using two-way ANOVA. Bracketed labels show between-group differences; bar-side labels show within-group canopy differences. 'ns' indicates no significant difference; asterisks indicate significance levels (* *p* < 0.05, ** *p* < 0.01, *** *p* < 0.001). Data are means ± SE (*n* = 6).

In the comparison between *P.b* and healthy *P.p*, PLC varied significantly with both variety and canopy position, and a significant interaction between these factors was also detected (*p* < 0.001; Fig. 3c). Upper canopy branches of *P.p* displayed markedly higher PLC than those of *P.b* (7.31 vs 1.56; *p* < 0.001). Within healthy *P.p* trees, PLC was substantially greater in the upper canopy than in the lower canopy (*p* < 0.001). In contrast, *P.b* exhibited the opposite pattern, with relatively lower PLC in the upper canopy. Hydraulic efficiency traits, including *K*_*l*_ and *K*_*s*_ were influenced by both variety and canopy position, and the interaction between these two factors was significant (*p* < 0.05; Fig. 3a, b). In *P.b*, both *K*_*l*_ and *K*_*s*_ were considerably higher in upper branches than in lower branches (*K*_*l*_: 9.78 vs 3.76; *p* < 0.05; *K*_*s*_: 4.80 vs 2.08; *p* < 0.001). By comparison, *P.p* showed no significant vertical variation in these hydraulic efficiency traits. Xylem anatomical characteristics, including WD, D, and VD, remained largely unchanged across varieties, canopy positions, and their interaction (*p* > 0.05; Fig. 3d−f).

Within *P.p* populations, comparisons between healthy and declining *P.p-D* revealed a significant interaction between health status and canopy position for PLC (*p* < 0.05; Fig. 3c). Declining *P.p-D* showed substantially higher PLC in upper canopy branches than healthy individuals (23.56 vs 7.31; *p* < 0.001). In both health conditions, PLC was significantly higher in the upper canopy than in the lower canopy (healthy: 7.31 vs 1.63; declining *P.p-D*: 23.56 vs 4.56). In contrast, hydraulic efficiency traits and xylem anatomical variables did not vary significantly with health status or canopy position, suggesting that the observed decline was mainly associated with differences in hydraulic safety rather than hydraulic efficiency or anatomical structure.

### Allometric relationships

**Figure 4:**
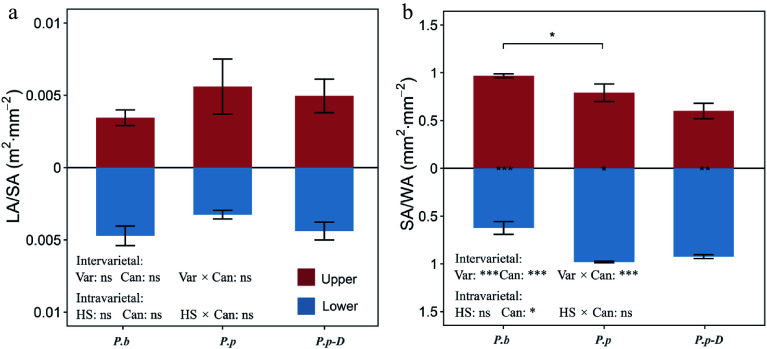
Variations in (a) LA/SA (leaf area to sapwood area ratio), an indicator of hydraulic supply–transpiration balance, and (b) SA/WA (sapwood area to whole cross-sectional wood area ratio), an indicator of functional xylem proportion, across different comparison groups: intervarietal (*P.b* vs *P.p*), intravarietal (healthy *P.p* vs declining *P.p-D*), and between upper and lower canopy positions within each group. The effects of variety (or health status) and canopy position were evaluated using two-way ANOVA. Bracketed labels show between-group differences; bar-side labels show within-group canopy differences. 'ns' indicates no significant difference; asterisks indicate significance levels (* *p* < 0.05, ** *p* < 0.01, *** *p* < 0.001). Data are means ± SE (*n* = 6).

The LA/SA was used to describe branch-level carbon allocation patterns (Fig. 4a). In the comparison between *P.b* and healthy *P.p*, LA/SA remained similar across varieties and canopy positions, and no interaction between these factors was detected (*p* > 0.05). In contrast, SA/WA, representing the proportion of functional sapwood, varied significantly with both variety and canopy position, and their interaction was significant (*p* < 0.001; Fig. 4b). Upper canopy branches of *P.b* had higher SA/WA values than those of *P.p* (*p* < 0.05). Moreover, *P.b* displayed a clear vertical pattern, with substantially greater SA/WA in upper branches than in lower branches (0.97 vs 0.62; *p* < 0.001). *P.p* showed the opposite trend, with reduced SA/WA values in the upper canopy (*p* < 0.05).

Within *P.p* populations, LA/SA did not vary significantly with tree health status, canopy position, or their interaction (*p* > 0.05). In contrast, SA/WA differed along the canopy gradient (*p* < 0.05), while health status and the interaction term were not significant (*p* > 0.05). Both healthy and declining *P.p-D* exhibited lower SA/WA values in the upper canopy than in the lower canopy (*p* < 0.01; Fig. 4b).

### Non-structural carbohydrate (NSC) traits

**Figure 5:**
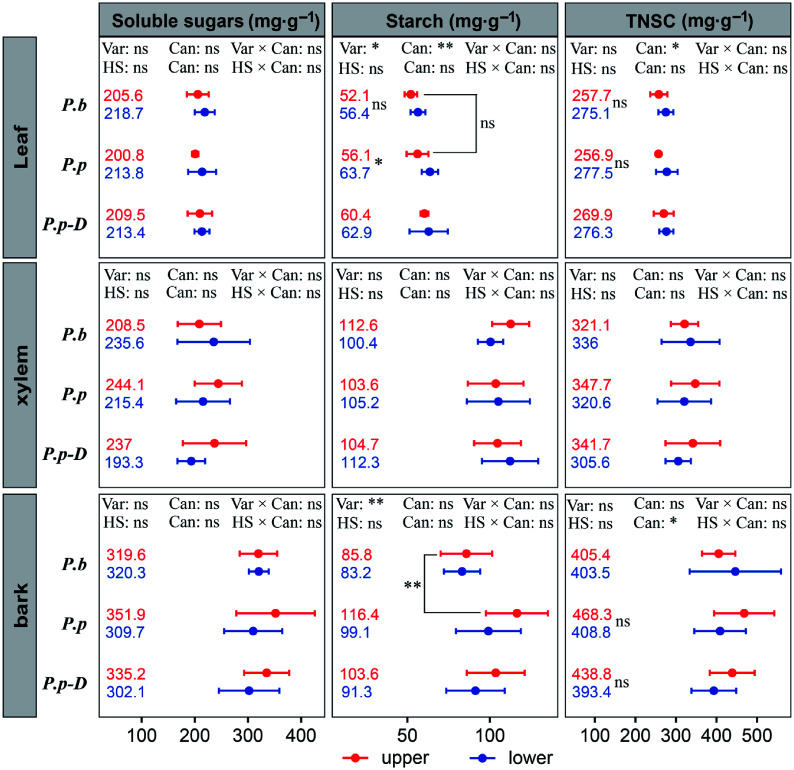
Variations in soluble sugars, starch, and total NSC in leaves, stems, and bark across different comparison groups: intervarietal (*P.b* vs *P.p*), intravarietal (healthy *P.p* vs declining *P.p-D*), and between upper and lower canopy positions within each group. The effects of variety (or health status) and canopy position were evaluated using two-way ANOVA. Bracketed labels show between-group differences; bar-side labels show within-group canopy differences. 'ns' marks non-significant differences; asterisks indicate significant differences (* *p* < 0.05, ** *p* < 0.01, *** *p* < 0.001). Data are means ± SE (*n* = 6).

Non-structural carbohydrate (NSC) concentrations varied across tissues, canopy positions, and varieties (Fig. 5). In the intervarietal comparison (*P.b* vs healthy *P.p*), leaf starch content was significantly influenced by both variety (*p* < 0.05), and canopy position (*p* < 0.01), with no significant interaction between these factors (*p* > 0.05). Within healthy *P.p* individuals, leaf starch content was significantly higher in the lower canopy than in the upper canopy (*p* < 0.01), whereas no such vertical difference was observed in *P.b* (*p* > 0.05). Leaf total NSC was affected only by canopy position (*p* < 0.05); however, post-hoc comparisons revealed no significant differences between the upper and lower canopy for either variety (*p* > 0.05). Bark starch content showed a significant intervarietal effect (*p* < 0.01), with *P.b* displaying lower values than *P.p*, particularly in the upper canopy. No other NSC components or tissues differed significantly between the two varieties (*p* > 0.05). In the intravarietal comparison between healthy *P.p* and declining *P.p-D*, only bark total NSC content was affected by canopy position (*p* < 0.05). Stratified comparisons, however, indicated no significant upper–lower canopy differences for either health status (*p* > 0.05). No other significant effects were detected (*p* > 0.05).

### Intervarietal and intravarietal radial growth patterns and CBA increment

Significant differences were detected in standardized RWI chronologies among *P.b*, healthy *P.p*, and declining *P.p-D* (Table 1). *P.p* exhibited a higher standard deviation (SD) and mean sensitivity (MS), indicating stronger climate signals. Declining *P.p-D* showed a higher signal-to-noise ratio (SNR), reflecting a more pronounced climate influence on growth. The first-order autocorrelation coefficient (AC1) reflects the lagged effect of the previous year's climate on current-year growth, with values of 0.073, 0.399, and 0.142 for *P.b*, *P.p*, and declining *P.p-D*, respectively. Among them, *P.p* displayed a relatively high AC1 coefficient, indicating that its growth is more strongly influenced by the previous year's climatic conditions. The mean correlation among all tree-ring series (R) measures the synchrony of RWI variations among trees within a chronology, with higher values indicating a stronger environmental signal. Declining *P.p-D* showed higher mean correlation within trees (R1) and between trees (R2) than both *P.p* and *P.b*. The expressed population signal (EPS) values were 0.859, 0.968, and 0.988, respectively, indicating that the collected samples sufficiently represent the local population.

**Table 1:** Key statistical characteristics of standardized chronologies.

Feature	*P.b*	*P.p*	*P.p-D*
Time interval	1975−2021	1974−2021	1974−2021
Mean sensitivity (MS)	0.156	0.313	0.294
Standard deviation (SD)	0.147	0.372	0.279
First-order autocorrelation coefficient (AC1)	0.073	0.399	0.142
Mean correlation among all radii (R)	0.253	0.517	0.721
Mean correlation within trees (R1)	0.248	0.537	0.665
Mean correlation between trees (R2)	0.252	0.507	0.738
Signal to noise ratio (SNR)	6.084	29.956	79.98
Expressed population signal (EPS)	0.859	0.968	0.988

**Figure 6:**
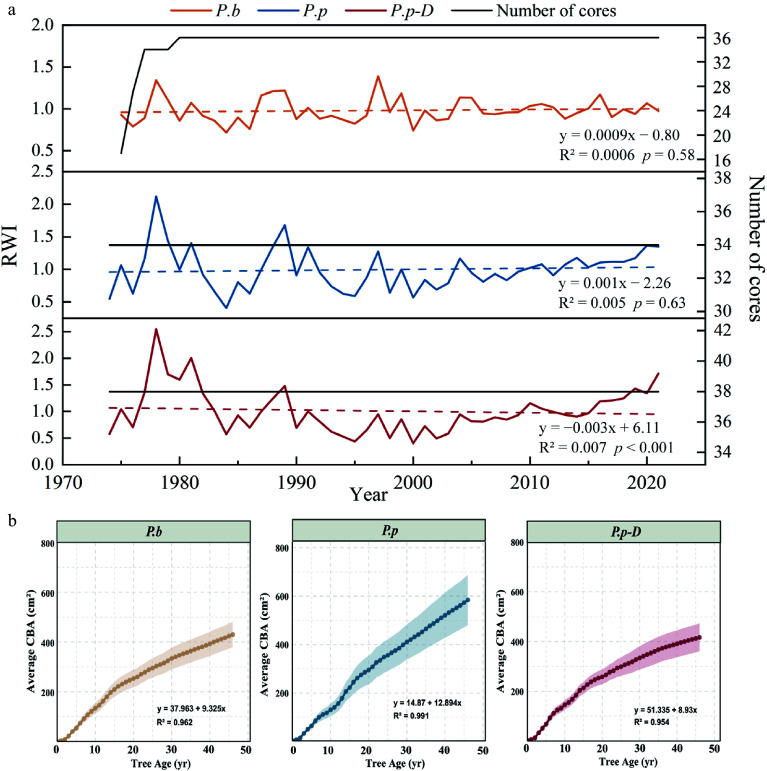
(a) Standardized tree-ring width index (RWI) chronologies for the three poplar groups. The black line indicates the number of cores available each year (right axis). (b) Average cumulative basal area increment (CBA) in relation to tree age for *P.b*, healthy *P.p*, and declining *P.p-D*. Shaded areas represent 95% confidence intervals, and solid lines represent regressions describing long-term growth trends.

RWI revealed significant differences in radial growth dynamics among *P.b*, healthy *P.p*, and declining *P.p-D* during 1974–2021 (Fig. 6a). *P.b* exhibited relatively small interannual fluctuations in RWI with an overall stable trend (R^2^ = 0.0006, *p* = 0.58), indicating long-term stability in radial growth. In contrast, *P.p* showed no significant long-term trend (R^2^ = 0.005, *p* = 0.63), but its RWI fluctuated more strongly, suggesting greater sensitivity to environmental variability. Notably, declining *P.p-D* exhibited a persistent decrease in RWI (slope = –0.003, R^2^ = 0.07, *p* < 0.001), reflecting a pronounced reduction in radial growth. Results from CBA further highlighted intervarietal and intravarietal growth differences (Fig. 6b). With increasing tree age, average CBA increased continuously in all three groups. However, *P.b* and *P.p* exhibited faster accumulation, with regression slopes of 9.325 and 12.894, respectively, reflecting stronger radial expansion capacity. In contrast, the growth rate of declining *P.p-D* was markedly lower (slope = 8.93), and its growth curve leveled off in later years, indicating restricted radial growth.

## Discussion

### Intervarietal differences in vertical hydraulic coordination underpin contrasting canopy dieback risk

Differences in canopy stability between the two *Populus* varieties under water-limited conditions are primarily associated with contrasting patterns of hydraulic safety regulation along the vertical canopy gradient rather than differences in overall hydraulic transport capacity. Our results suggest that the distinct drought responses of *P.b* and *P.p* are largely determined by the distribution of hydraulic tension and embolism risk within the crown, particularly in upper canopy branches^[10]^. Although *Ψ*_pd_ varied significantly with both variety and canopy position, no clear intervarietal difference was observed in the upper canopy. This finding implies that predawn water status alone cannot fully explain the contrasting drought responses between the two varieties^[4,42]^. In contrast, *Ψ*_md_ was consistently more negative in *P.p*, especially in the upper canopy, indicating that these branches experienced greater hydraulic tension under high atmospheric demand. Together with the substantially higher PLC observed in the upper canopy branches of *P.p*, this pattern indicates an elevated risk of embolism formation under drought conditions. However, increased embolism vulnerability does not necessarily trigger immediate stomatal closure. Some *Populus* species maintain relatively high stomatal conductance even at low water potentials, thereby sustaining carbon assimilation at the expense of hydraulic safety^[12,16]^. Such a hydraulic strategy has frequently been associated with drought-induced branch dieback^[43,44]^.

Despite the absence of consistent intervarietal differences in branch hydraulic efficiency (*K*_*l*_ and *K*_*s*_) across canopy positions, the significant interaction between variety and canopy position revealed contrasting patterns of vertical hydraulic coordination. In *P.b*, upper canopy branches maintained relatively low PLC while preserving hydraulic conductivity, suggesting coordinated adjustments that help maintain functional xylem in hydraulically exposed portions of the crown^[45,46]^. This interpretation is further supported by the higher SA/WA observed in the upper canopy of *P.b*, indicating preferential sapwood that may help buffer embolism formation under drought stress^[36]^. In contrast, *P.p* exhibited weaker vertical coordination of hydraulic traits. Upper canopy branches experienced more negative Ψ_md_ and higher PLC, yet these changes were not accompanied by corresponding increases in hydraulic efficiency or sapwood allocation. Such a mismatch between hydraulic demand and structural adjustment may facilitate embolism accumulation in exposed branches, plausibly explaining why *P.p* is more susceptible to canopy degradation and branch dieback under prolonged drought^[43,47]^. Similar top-down patterns of canopy decline linked to stronger hydraulic limitation in upper branches have also been reported in drought-stressed *Populus* species^[19]^, highlighting the importance of within-crown hydraulic gradients in determining drought vulnerability.

Canopy architecture may further amplify these intervarietal differences by shaping vertical gradients in light interception and transpirational demand. Field observations showed that *P.b* tends to form a broader and more layered crown, whereas *P.p* typically develops a narrow, columnar canopy. A wider crown may reduce peak transpiration and embolism risk by distributing leaf area more evenly across canopy layers^[48−50]^, thereby stabilizing branch water potential. In contrast, the columnar crown structure of *P.p* may concentrate transpiration in upper canopy branches and lengthen hydraulic transport pathways, increasing hydraulic strain in exposed tissues. This interpretation is consistent with recent findings suggesting that columnar *Populus* crowns may intensify hydraulic limitation during drought^[50]^. Compared with hydraulic traits, carbon-related variables showed relatively limited variation among varieties and canopy positions. Although *P.p* displayed higher starch concentrations in certain tissues, total NSC levels remained broadly comparable among groups. This pattern suggests that the observed branch dieback was unlikely to result primarily from carbon depletion. Instead, the results support previous studies indicating that hydraulic failure often precedes significant NSC depletion during drought-induced mortality in *Populus*^[12,51,52]^.

### Hydraulic dysfunction in upper canopy branches underlies growth decline in *Populus alba* var. *pyramidalis*

Hydraulic constraints appear to play a central role in driving growth decline and branch mortality in *P.p* under prolonged water stress^[43]^. In this variety, canopy dieback and reduced growth were primarily associated with localized hydraulic dysfunction in upper canopy branches rather than with a uniform decline in whole-tree hydraulic transport capacity. Healthy *P.p* and declining *P.p-D* were exposed to similar irrigation regimes and groundwater conditions. Correspondingly, *Ψ*_pd_ did not differ significantly between health states or across canopy positions (Fig. 2a), indicating similar baseline water status among individuals. This observation suggests that the observed decline is unlikely to be explained by persistent differences in soil water access or predawn plant water status^[4,53,54]^. In contrast, PLC displayed a pronounced interaction between health status and canopy position. Declining *P.p-D* exhibited substantial embolism accumulation in upper canopy branches, whereas lower canopy branches were comparatively less affected. At the same time, branch-level hydraulic efficiency (*K*_*l*_ and *K*_*s*_) and xylem anatomical traits showed little variation across health states or canopy positions (Fig. 3a, c). These results indicate that canopy decline in *P.p* reflects spatially localized hydraulic dysfunction rather than a general reduction in conductive capacity. Such spatial concentration of hydraulic impairment is consistent with previous observations in drought-stressed *Populus* species and other diffuse-porous trees, which often display high vulnerability to embolism formation^[12,16,46]^. The greater hydraulic strain experienced by upper branches likely results from the combined effects of longer hydraulic path length and stronger atmospheric demand along the canopy gradient. Under conditions of high light exposure and elevated vapor pressure deficit, these factors can disproportionately increase embolism risk in exposed branches^[45,48]^. Similar patterns have been reported in several *Populus* species, where upper canopy branches accumulate embolism more readily under drought stress, particularly in fast-growing diffuse-porous taxa^[12,19]^. This mechanism provides a physiological explanation for the frequently observed top-down progression of canopy dieback, where distal branches fail before whole-tree hydraulic collapse^[50]^.

Changes in biomass allocation may further contribute to this process. Declining *P.p-D* showed lower SA/WA ratios and greater variability in this trait (Fig. 4b), suggesting reduced proportional investment in functional sapwood relative to supporting tissues. Such shifts in allocation could further compromise hydraulic safety and accelerate functional decline^[36,55]^. Despite clear evidence of hydraulic impairment, total NSC concentrations remained broadly similar across canopy positions and between healthy and declining trees, with no indication of large-scale carbon depletion. This pattern reinforces the view that hydraulic dysfunction and carbon starvation can become decoupled during drought stress, with hydraulic limitation acting as the primary driver of decline in this system^[12]^. Nevertheless, carbon allocation strategies may still influence drought responses. Higher LA/SA or SA/WA has the potential to buffer water stress under certain conditions^[36]^. In the present study, however, the reductions in SA/WA observed in declining *P.p-D* were insufficient to offset hydraulic dysfunction, as reflected in elevated PLC and reduced radial growth.

### Short-term hydraulic traits and long-term tree-ring characteristics indicate growth-safety trade-offs

Previous studies have shown that species with higher xylem conductivity often achieve faster radial growth^[56]^. A similar pattern emerged in the present study. Healthy *P.p* individuals exhibited larger mean DBH than both *P.b* and declining *P.p-D*, consistent with their higher radial growth rates. Differences in radial growth among the three groups corresponded closely to variation in hydraulic traits (Figs 3, 6). For example, healthy *P.p* displayed the highest CBA growth rate (slope = 12.894), which coincided with relatively high hydraulic efficiency (*K*_*l*_ and *K*_*s*_). However, this growth advantage was accompanied by lower hydraulic safety. In contrast, *P.b* exhibited a more balanced strategy: radial growth was moderate (CBA slope = 9.325), while PLC remained comparatively low, indicating greater hydraulic safety^[44,57]^. The elevated *K*_*l*_ observed in upper canopy branches of *P.b* likely enhances water delivery to leaves during periods of high atmospheric demand, thereby helping to maintain stable growth over time. Declining *P.p-D* showed a markedly lower CBA growth rate (slope = 8.93) and a clear slowdown in growth during later stages. Despite having a stand age comparable to healthy *P.p*, these trees displayed smaller DBH values together with substantially higher PLC in upper canopy branches (Figs 3c, 6b). The concentration of embolism in these branches suggests that hydraulic constraints progressively limit water transport. Importantly, this limitation appears to arise from embolism accumulation rather than a general reduction in hydraulic efficiency, since *K*_*l*_ and *K*_*s*_ did not differ significantly between healthy and declining trees. Similar patterns have been reported in other studies, where drought-induced embolism accumulation suppresses long-term radial growth without necessarily reducing theoretical conductivity^[16,58,59]^.

Tree-ring characteristics further support the link between short-term hydraulic impairment and long-term growth dynamics. In declining *P.p-D*, the higher PLC observed in upper canopy branches likely reduced hydraulic safety and constrained gas exchange during drought periods, effects that are subsequently reflected in multi-year growth chronologies^[42,60]^. Chronological statistics revealed that healthy *P.p* had higher SD, MS, and AC1 values (Table 1), indicating stronger sensitivity to climatic variability and continued dependence of growth on previous year's water availability^[3]^. By contrast, declining *P.p-D* showed higher SNR and R values, suggesting increasingly synchronized growth responses among trees exposed to water limitation. Such synchronization likely reflects reduced physiological buffering capacity against climatic stress. These patterns align with the variation in branch hydraulic traits and CBA trends, indicating that short-term hydraulic impairment and long-term reductions in radial growth are closely linked. Over time, the cumulative effects of hydraulic limitation may contribute to the top-down progression of canopy dieback observed in susceptible individuals^[45]^. In irrigated shelterbelt plantations, this process may be further exacerbated when irrigation supply becomes insufficient^[24]^. Integrating long-term tree-ring records with hydraulic trait measurements, therefore provides a valuable framework for understanding how hydraulic processes shape drought-induced growth decline.

## Conclusions

This study provides evidence that drought-induced decline in poplar shelterbelt plantations of two *Populus alba* varieties (*P.b* and *P.p*) is closely associated with canopy-scale hydraulic dysfunction rather than carbon depletion. Spatially heterogeneous hydraulic failure within the canopy, driven by divergent plastic responses of branch functional traits in the two varieties along the vertical canopy gradient represents a key mechanism linking drought stress to growth decline and top-down canopy dieback. By revealing growth and hydraulic safety trade-offs among these closely related poplar varieties, our findings highlight the critical role of vertical hydraulic coordination in determining drought vulnerability, advancing understanding beyond traditional whole-tree perspectives. Future research integrating long-term hydraulic and carbon dynamics will further improve predictions for shelterbelt plantation resilience under intensifying drought.

## Data Availability

The datasets generated during and/or analyzed in the current study are available from the corresponding author on reasonable request.
